# Liver X Receptor Activation Alleviates Hepatic Ischemia‐Reperfusion Injury in Diabetes by Inhibiting NF‐κB–NLRP3 Activation

**DOI:** 10.1002/iid3.70243

**Published:** 2025-08-08

**Authors:** Chuanwei Jiang, Wenzhou Ding, Yuanchang Hu, Chao Yang

**Affiliations:** ^1^ Hepatobiliary Center The First Affiliated Hospital with Nanjing Medical University Nanjing China; ^2^ Key Laboratory of Liver Transplantation Chinese Academy of Medical Sciences Nanjing China; ^3^ NHC Key Laboratory of Hepatobiliary Cancers Nanjing China

**Keywords:** diabetes, liver ischemia‐reperfusion injury, LXR, macrophage

## Abstract

**Introduction:**

Hyperglycemia has been reported to be a crucial factor that aggravates liver ischemia‐reperfusion injury (IRI). Macrophage–mediated inflammatory injury is vital to liver IRI. A positive effect of Liver X receptor (LXR) on diabetes has been proven; however, the function and mechanism of LXR in diabetic liver IRI remain unclear. Accordingly, our study concentrates on the mechanism underly.

**Materials and Methods:**

Streptozotocin (STZ, 40 mg/kg)‐treated diabetic mice were used to establish a liver IRI model. Bone marrow–derived macrophages (BMDMs) were used in studying the role of macrophage inflammation in diabetic liver. GW3965 hydrochloride was used to activate LXR in vivo and in vitro. QD394, a lipid peroxidation agonist, was used to verify the underlying mechanism.

**Results:**

Hyperglycemia exacerbates liver ischemia‐reperfusion injury (IRI) by promoting hepatic cell death and inflammation in vivo. In diabetic livers, the expression of liver X receptors (LXRs) is significantly reduced. Furthermore, the ischemia‐reperfusion process itself further decreases LXR levels. Application of the LXR agonist GW3965 mitigates macrophage lipid peroxidation and inflammasome NLRP3 (NOD‐like receptor thermal protein domain associated protein 3) inflammasome activation in vitro, thereby protecting the liver from severe IRI. The results were further confirmed by the rescue experiments.

**Conclusions:**

LXRs play an important role in diabetic liver IRI and macrophage‐associated inflammation. Pharmacologic activation of LXRs alleviates macrophage inflammatory activation in diabetic liver IRI, and may serve as a potential therapeutic target for diabetes‐related liver injury.

## 1 Introduction

Epidemiological studies have shown an increasing incidence of diabetes mellitus worldwide [1]. 589 million adults (20–79 years) are living with diabetes worldwide reported by The Diabetes Atlas from International Diabetes Federation [2]. The state of hyperglycemia leads to numerous complications and exacerbates organ ischemia‐reperfusion injury (IRI) [3, 4]. We previously found that hyperglycemia aggravates acute liver injury via the ATF6 (Activating Transcription Factor 6)‐CHOP (C/EBP‐homologous protein) pathway [5]. However, the mechanism by which diabetes exacerbates liver IRI remains unclear.

Liver IRI is a critical process that occurs during liver resection and transplantation [6]. The innate immune system plays an important role in this sterile inflammatory injury [7]. Macrophages, a vital part of innate immunity, have been reported to contribute to many types of acute liver injury including liver IRI and drug‐induced liver injury [8, 9, 10]. In the liver IRI process, hepatocyte damage and macrophage‐related inflammation are mutually reinforcing [11]. Hyperglycemia may exacerbate hepatocyte damage and activate macrophage‐triggered inflammation.

Liver X receptors (LXRs) are associated with cholesterol accumulation [12] and diabetes. It has been reported that lower expression of the LXR pathway is a major risk factor for type 2 diabetes [13]. LXR signaling has also been reported to regulate macrophage inflammation in response to ionizing radiation [14]. LXRs are also related to the synthesis of anti‐inflammatory fatty acid in macrophages [15]. Activation of LXRs could alleviate diabetes‐induced systemic dysfunction [16]. In the diabetic liver, hyperglycemia may impair LXR function and exacerbate liver injury. Elevated glucose levels induce oxidative and endoplasmic reticulum (ER) stress [17], damaging LXR proteins and disrupting their signaling. Additionally, epigenetic changes and mitochondrial dysfunction further compromise LXR function [18]. LXR signaling could alleviate alcoholic liver injury by suppressing ER stress [19]. However, whether LXRs play a vital role in diabetic liver IRI and the mechanism underlying this remains unknown.

In our current research, we studied LXR expression in diabetic liver IRI. Aminotransferase alanine aminotransferase (ALT) and aspartate aminotransferase (AST), hematoxylin and eosin (H&E) staining and cell death‐related proteins were detected to judge liver ischemia reperfusion injury. NLRP3, NF‐κB and cytokine assays were detected to estimate macrophage associated inflammation level. Superoxide dismutase (SOD) and malondialdehyde (MDA) were tested to study whether lipid peroxidation plays vital role in diabetic liver IRI and under pharmacologic activation of the LXR expression.

## 2 Materials and Methods

### 2.1 Methods Statement

All methods were performed in accordance with the relevant guidelines and regulations. All animal experiment procedures met the relevant legal and ethical requirements according to the protocol (2024‐SRFA‐649) endorsed by the Institutional Animal Care and Use Committee of Nanjing Medical University in accordance with the ARRIVE guidelines.

### 2.2 Animals and Models

8‐week‐old male C57BL/6 J mice (weight 20–24 g) were purchased from GemPharmatech Co. Ltd. The mice had free access to food and water under a 12 h light/dark cycle in temperature 23 ± 3°C. After 1 week of acclimatization, Streptozotocin (STZ, 40 mg/kg, MedChemExpress, HY‐13753) or vehicle control was intraperitoneally injected into mice for 5 days, and a blood glucose concentration over 300 mg/dL on day 14 was considered indicative of diabetes [5].

A classic 70% liver IRI model was conducted [20]. After successful anesthesia with 1.5% isoflurane (RWD Life Science, cat. R510‐22‐10), an elastic microvascular clamp was used to block the arterial and portal venous blood supply to the cephalic lobes (70%) of the whole liver for 90 min. Then, the atraumatic clip was removed, and liver reperfusion was performed for 6 h. Liver and blood samples were harvested after CO_2_ inhalation euthanasia. Five mice were used in each experimental group.

To conduct the rescue experiment, GW3965 (MedChemExpress, cat. HY‐10627A), the agonist of LXR, was injected intraperitoneally at a dose of 10 mg/kg [21] 24 h before modeling. QD394 (MedChemExpress, cat. HY‐139369), a lipid peroxidation activator, was injected intraperitoneally at a dose of 10 mg/kg [22] one dose 4 h before the liver IRI model.

### 2.3 Disposal of Liver Tissue

Liver specimens were fixed in 4% paraformaldehyde and embedded in paraffin for H&E and immunohistochemistry (IHC) staining. The Suzuki score (congestion 0–4, vacuolation 0–4, necrosis 0–4) was used to measure liver injury.

### 2.4 Bone Marrow–Derived Macrophages (BMDMs) Isolation and Culture

BMDMs were obtained from the bone marrow of mouse femurs and tibias. The cells were cultured in high glucose(30 mM) DMEM (ThermoFisher Scientific, cat. 11965118) with 10% FBS (ThermoFisher Scientific, cat. A3160902), 1% P/S (ThermoFisher Scientific, cat. 15140122), and 1% HEPES (ThermoFisher Scientific, cat. 15630080) for 7 days [23].

Lipopolysaccharide (LPS, MedChemExpress, cat. HY‐D1056) was added to the culture medium at a concentration of 1 µg/ml for 24 h to activate BMDMs. In some groups, GW3965 was added at a concentration of 10 µM 2 h before LPS for mechanism research.

### 2.5 Serum Transaminase Examination

Serum concentrations of ALT and AST were quantified using an AU680 clinical chemistry analyzer (Beckman Coulter).

### 2.6 Western Blot

The concentration of tissue or cell protein was measured using a bicinchoninic acid protein assay kit (Beyotime, cat. P0012). Proteins were subjected to SDS‐PAGE and transferred onto a polyvinylidene 550 fluoride nitrocellulose membrane. Antibodies against cleaved caspase‐3 (1:1000, Cell Signaling Technology, cat. #9661S), cleaved caspase‐1 (1:1000, Cell Signaling Technology, cat. #89332S), BAX (1:500, Cell Signaling Technology, cat. #5023 T), Bcl‐2 (1:1000, Cell Signaling Technology, cat. #3498S), LXR‐α (1:2000, ABclonal, cat. A3974), LXR‐β (1:500, ABclonal, cat. A22545), NLRP3 (1:2000, ABclonal, cat. A24294), NF‐κB (1:1000, ABclonal, cat. A19653) and β‐actin (1:20000, ABclonal, cat. AC004) were incubated at 4°C overnight. Secondary antibodies conjugated with HRP (1:2000, Cell Signaling Technology, cat. 7074P2) were then incubated at room temperature for 2 h. Finally, ECL Ultra Western HRP substrate (NCM Biotech, cat. P2300) was applied, and images were acquired using a Vilber chemiluminescent imaging system.

### 2.7 Quantitative RT‐PCR

Total RNA was extracted using TRIzol reagent (ThermoFisher Scientific, cat. 15596026CN), and quantified by Nanodrop One (ThermoFisher Scientific), followed by reverse transcription into cDNA using HiScript II Q RT SuperMix for qPCR (Vazyme, cat. R223‐01). Quantitative real‐time polymerase chain reaction (qRT‐PCR) was carried out on the QuantStudio 5 Real‐Time PCR System (ThermoFisher Scientific) with cDNA in triplicate using ChamQ SYBR qPCR Master Mix (Vazyme, cat. Q711‐02). The target gene expression was normalized against that of β‐actin. The primers were as follows: Tumor necrosis factor (TNF‐α) (F: 5′‐CAGGCGGTGCCTATGTCTC‐3′; R: 5′‐CGATCACCCCGAAGTTCAGTAG‐3′), IL‐1β (F: 5′‐GAAATGCCACCTTTTGACAGTG‐3′; R: 5′‐TGGATGCTCTCATCAGGACAG‐3′), IL‐6 (F: 5′‐TAGTCCTTCCTACCCCAATTTCC‐3′; R: 5′‐TTGGTCCTTAGCCACTCCTTC‐3′), IL‐18 (F: 5′‐GACTCTTGCGTCAACTTCAAGG‐3′; R: 5′‐CAGGCTGTCTTTTGTCAACGA‐3′), β‐actin (F: 5′‐TGACGTGGACATCCGCAAAG‐3′; R: 5′‐CTGGAAGGTGGACAGCGAGG‐3′).

### 2.8 Lipid Peroxidation Detection

The Total Superoxide Dismutase Assay Kit with WST‐8 (Beyotime, cat. S0101S) and Lipid Peroxidation MDA Assay Kit (Beyotime, cat. S0131S) were used according to the manufacturer's protocols to measure lipid peroxidation.

### 2.9 Statistical Analysis

All data are presented as the mean ± standard deviation based on at least three independent experiments. The Student's *t*‐test and one‐way analysis of variance (ANOVA) were used to analyze the differences among different groups. All the *p*‐values were two‐sided, and *p* < 0.05 indicated statistical significance (*p < 0.05, **p < 0.01, ***p < 0.001, and ****p < 0.0001).

## 3 Results

### 3.1 Hyperglycemia Aggravated Liver IRI

**Figure 1:**
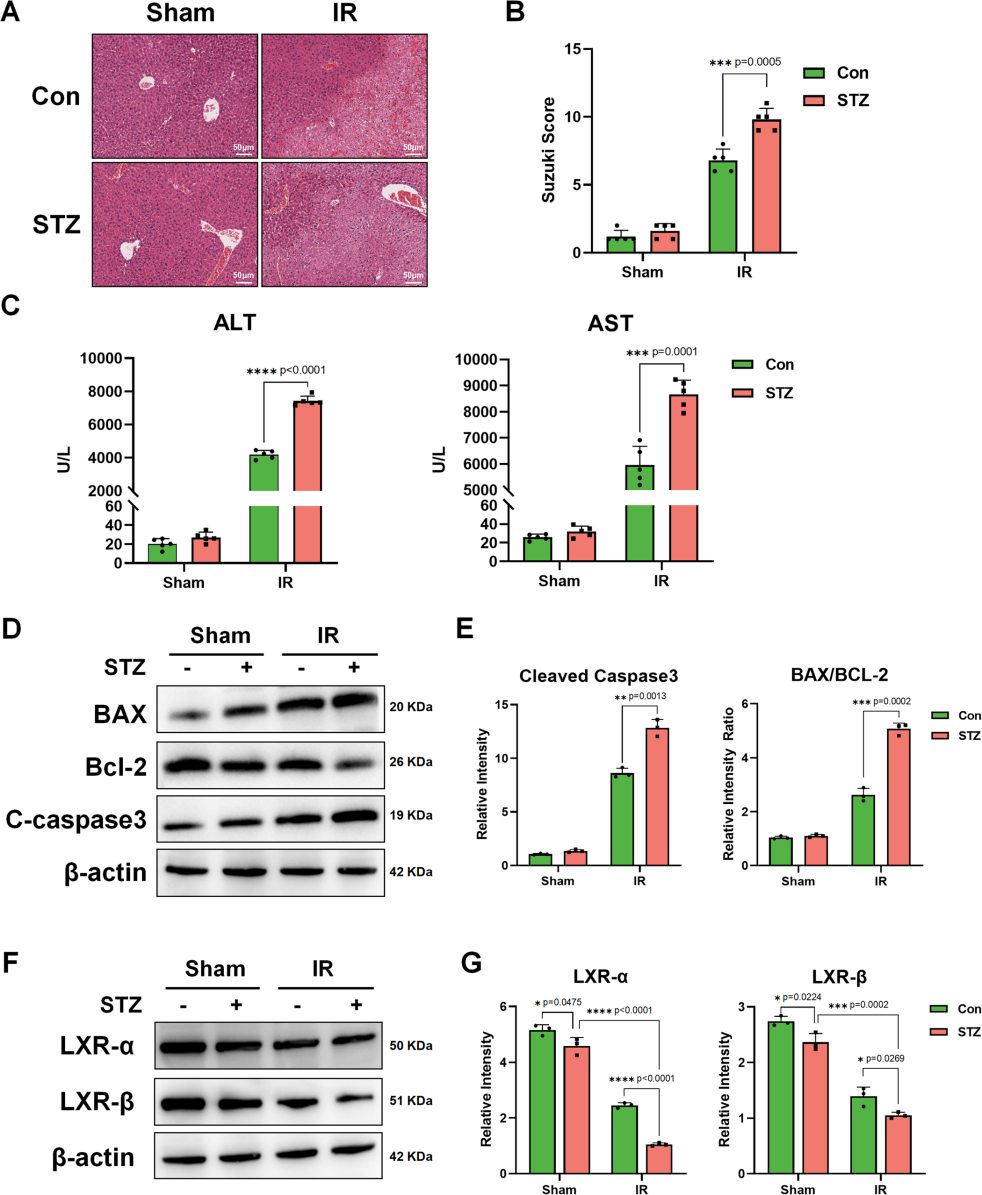
Hyperglycemia aggravated liver ischemia reperfusion injury. Diabetes C57BL/6 mice induced by STZ and control mice were subjected to 70% liver IRI model as methods described (*n* = 5/group). (A). Representative H&E staining of liver tissue (scale bar: 50 μm) (B). Suzuki Score of different groups (*n* = 5/group). (C). ALT and AST in mice serum were tested (*n* = 5/group). (D). Western Blot detection of BAX, Bcl‐2, cleaved caspase‐3 (C‐caspase3), and β‐actin in liver tissue. (E). Data statistics of WB strips gray value obtained by ImageJ (*n* = 3/group). (F). Western Blot detection of LXR‐α, LXR‐β, and β‐actin in liver. (G). Data statistics of WB strips gray value obtained by ImageJ (*n* = 3/group). *p < 0.05, **p < 0.01, ***p < 0.001, and ****p < 0.0001. *p*‐values were marked on the graphs.

Firstly, we used STZ‐induced diabetic mice to construct a classic 70% liver ischemia‐reperfusion model [5]. H&E staining of liver sections (Figure 1A) and Suzuki scores (Figure 1B) showed that STZ‐induced diabetes aggravated liver IRI. We also measured plasma transaminase levels after IR or sham operation and found that ALT and AST levels in STZ‐IR group were significantly higher than those in the control‐IR group (Figure 1C). Western Blot analysis also showed increased expression of BAX and cleaved caspase‐3, which means more cell death, in the STZ‐IR group (Figure 1D,E). These results confirm that diabetes aggravates liver injury and cell death post‐IR.

### 3.2 LXR Agonist Alleviated Diabetic Liver IRI In Vivo

LXRs, including LXR‐α and LXR‐β, are well known for playing a crucial role in the regulation of lipid metabolism and cholesterol homeostasis. LXR is also a glucose sensor and is found at lower levels in the diabetic body [13].

**Figure 2:**
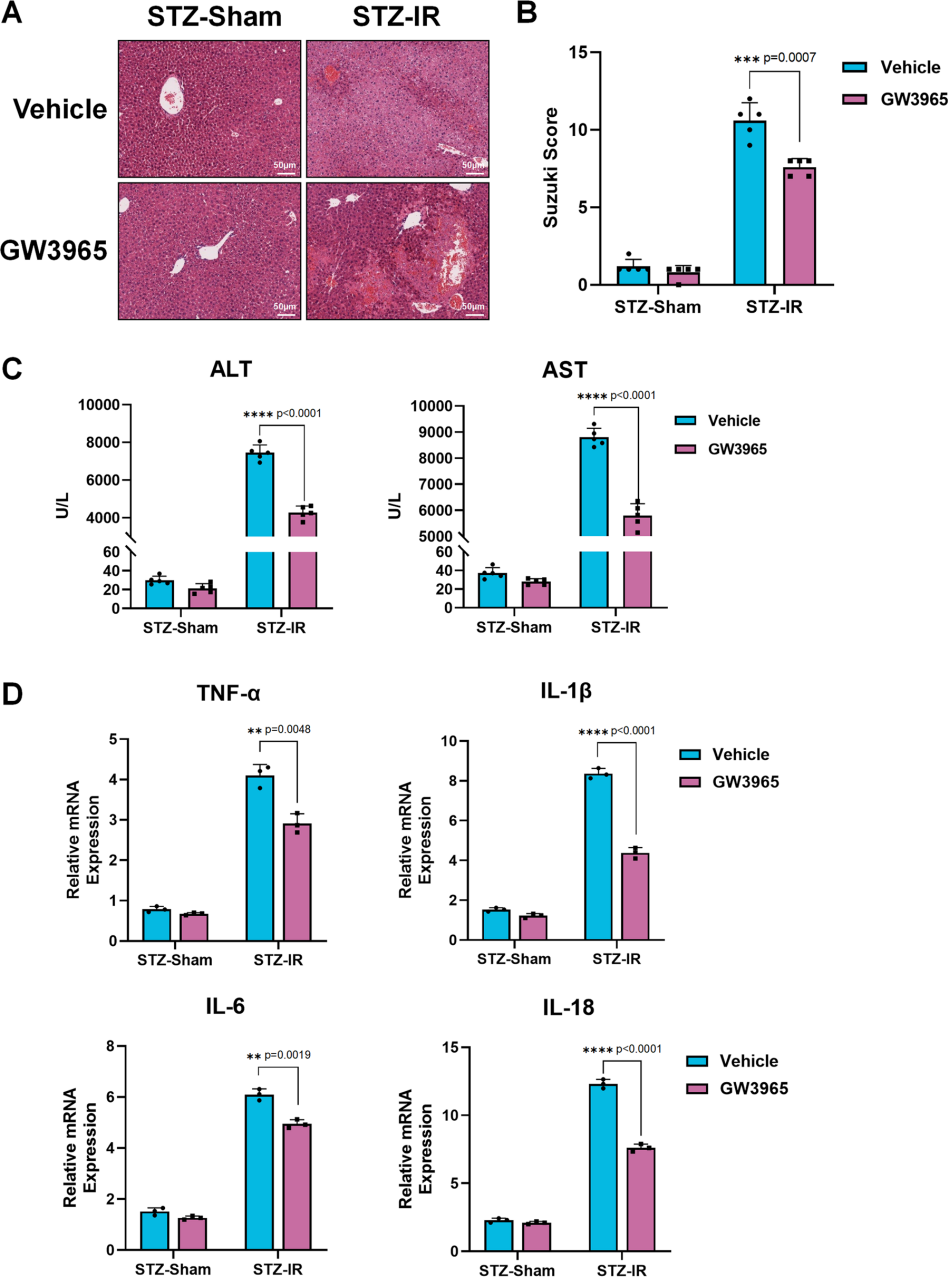
LXR agonist alleviated diabetic liver IRI in vivo. Diabetes C57BL/6 mice induced by STZ were pretreated with GW3965 or phosphate buffered saline (PBS) (Vehicle) before subjected to 70% liver IRI model (*n* = 5/group). (A). Representative H&E staining of liver tissue (scale bar: 50 μm) (B). Suzuki Score of different groups (*n* = 5/group). (C). ALT and AST in mice serum were tested (*n* = 5/group). (D). Gene expression of TNF‐α, IL‐1β, IL‐6, and IL‐10 relative to GAPDH in liver tissues measured by qRT‐PCR (*n* = 3/group). *p < 0.05, **p < 0.01, ***p < 0.001, and ****p < 0.0001. *p*‐values were marked on the graphs.

Thus, we tested LXR‐α and LXR‐β expression in the liver before and after ischemia reperfusion (IR). Western blot showed that both LXR‐α and LXR‐β expression were lower in diabetic liver, and liver IR further reduced their expression (Figure 1F,G). Therefore, we used GW3965, an LXR agonist, in vivo to pretreat STZ‐induced diabetic mice, and reconstruct liver IRI model again. Activation of LXR alleviates liver IRI, as demonstrated by H&E staining and Suzuki scores (Figure 2A,B). The decreased levels of ALT and AST in GW3965 group post‐IR also further supported this finding (Figure 2C).

### 3.3 LXR Activation Suppressed Macrophage‐Associated Inflammation via NLRP3–NF‐κB in Hyperglycemic Liver IRI

**Figure 3:**
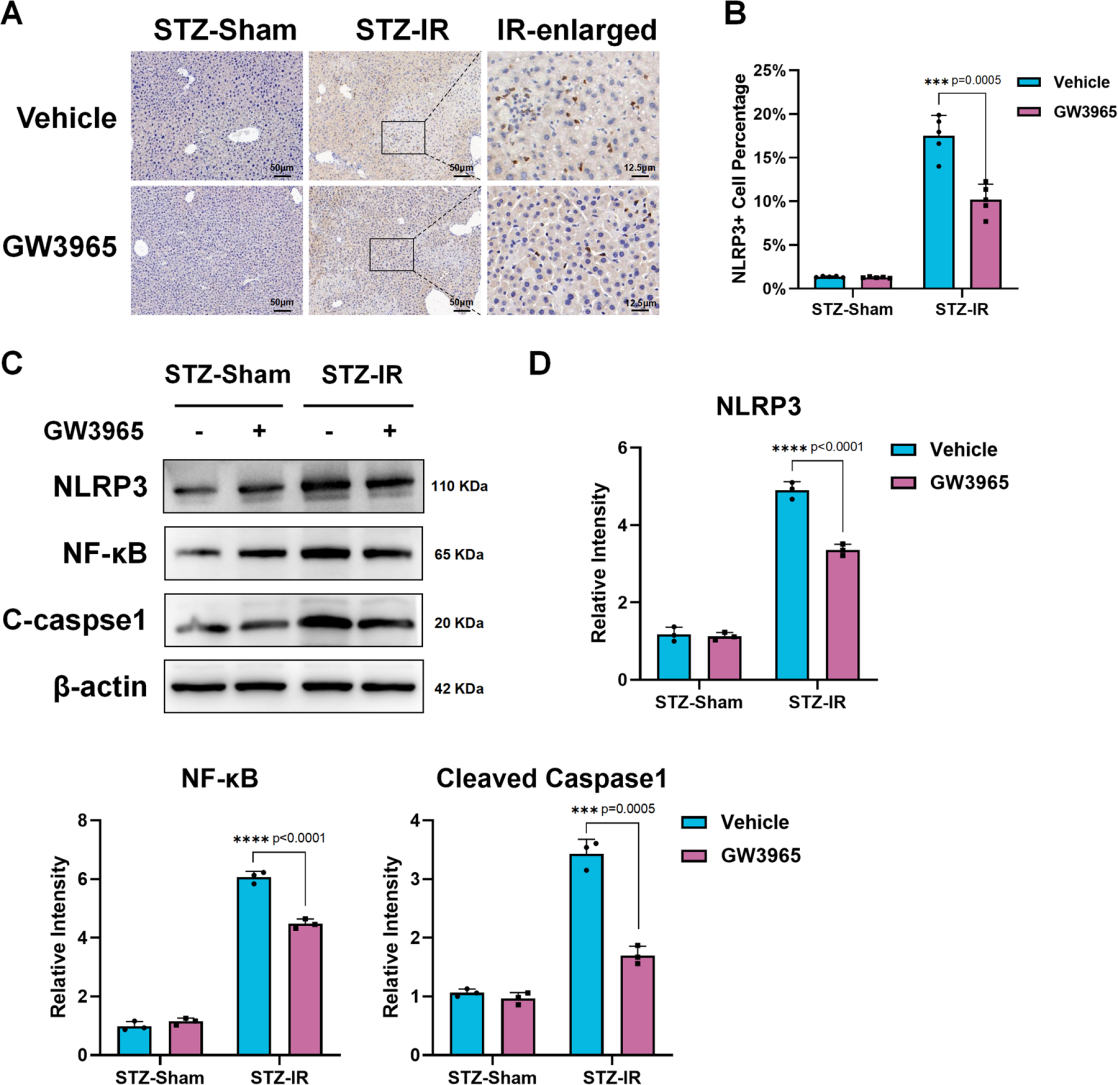
LXR activation suppressed hepatic inflammation via NLRP3–NF‐κB in hyperglycemia liver IRI. Diabetes C57BL/6 mice induced by STZ were pretreated with GW3965 or PBS (Vehicle) before subjected to 70% liver IRI model (*n* = 5/group). (A). Representative IHC staining of NLRP3 in liver tissue and enlarged images (scale bar: 50 μm or 12.5 μm) (B). Data statistics of NLRP3+ cells in liver tissue IHC staining images (*n* = 5/group). (C). Western Blot detection of NLRP3, NF‐κB, cleaved caspase‐1 (C‐caspase1), and β‐actin in liver tissue (*n* = 3/group). (D). Data statistics of WB strips gray value obtained by ImageJ (*n* = 3/group). *p < 0.05, **p < 0.01, ***p < 0.001, and ****p < 0.0001. *p*‐values were marked on the graphs.

LXR is associated with the innate immune system and inflammatory responses [24]. Therefore, we began with in vivo experiments. qPCR analysis of liver tissue RNA showed decreased levels of the pro‐inflammatory cytokines TNF‐α, IL‐1β, IL‐6, and IL‐18 in GW3965 group post IR (Figure 2D). It is worth noting that the difference in IL‐1β and IL‐18 expression (both downstream of NLRP3) was more pronounced than TNF‐α and IL‐6, suggesting potential difference in NLRP3 activation. Therefore, we performed IHC staining for NLRP3. The results showed that GW3965 treatment reduced NLRP3‐positive cells in the diabetic liver post‐IR (Figure 3A,B). Western blot analysis also showed decreased protein expression of NLRP3, NF‐κB, and cleaved caspase‐1 in the GW3965 group post‐IR (Figure 3C,D).

**Figure 4:**
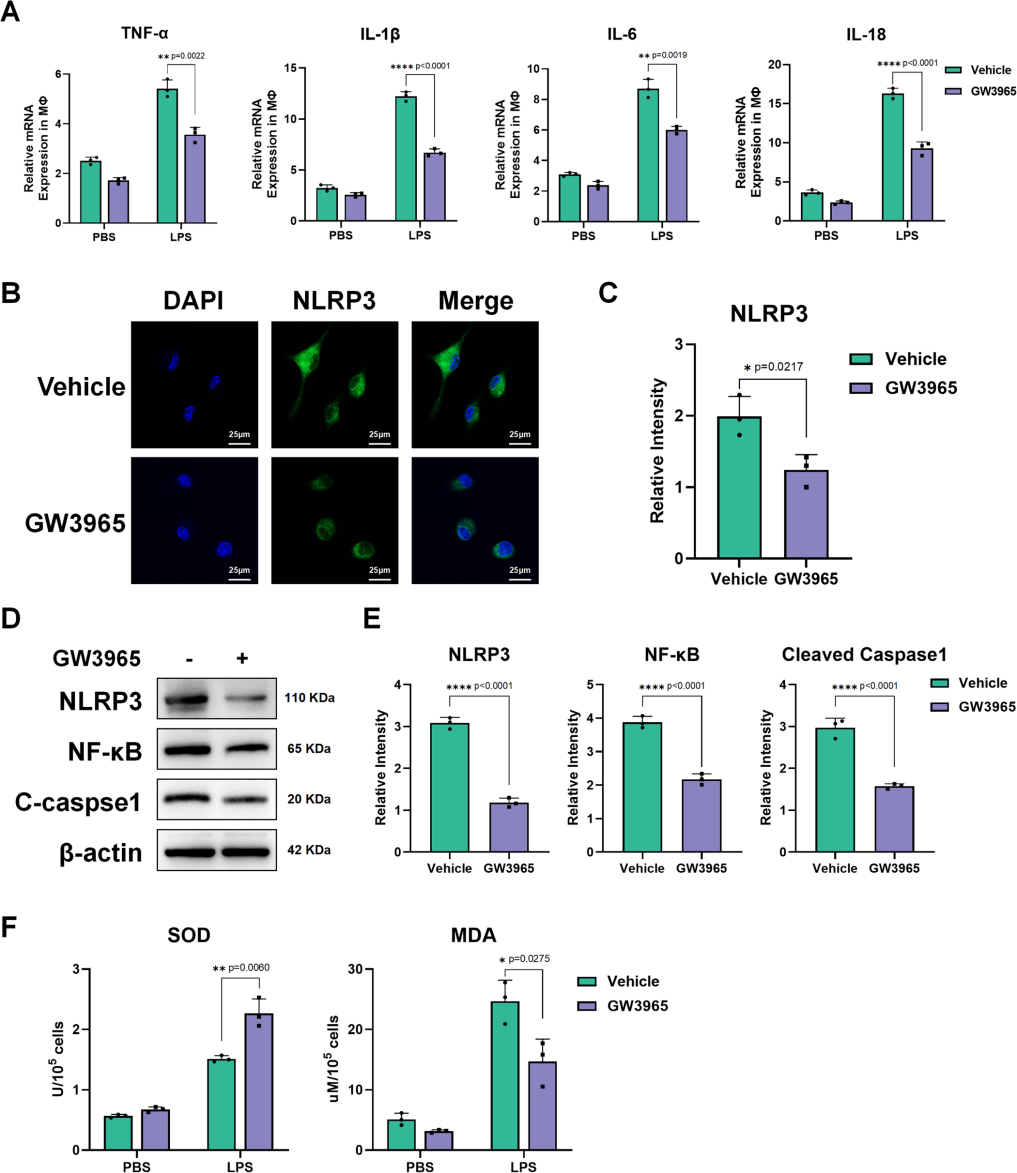
LXR activation suppressed macrophage‐mediated inflammation via NLRP3–NF‐κB in vitro. Mature bone marrow derived macrophages (BMDMs) were pretreated with GW3965 or PBS (vehicle) before LPS stimulation as methods described (*n* = 3/group). (A). Gene expression of TNF‐α, IL‐1β, IL‐6, and IL‐10 relative to GAPDH in BMDMs measured by qRT‐PCR (*n* = 3/group). (B). Representative IF staining of NLRP3 and DAPI in BMDMs and merged images (*n* = 3/group) (scale bar: 25 μm) (C). Data statistics of relative intensity of NLRP3 in BMDMs (*n* = 3/group). (D). Western Blot detection of NLRP3, NF‐κB, cleaved caspase‐1 (C‐caspase1), and β‐actin in BMDMs (*n* = 3/group). (E). Data statistics of WB strips gray value obtained by ImageJ (*n* = 3/group). (F). SOD and MDA levels of BMDMs (*n* = 3/group). *p < 0.05, **p < 0.01, ***p < 0.001, and ****p < 0.0001. *p*‐values were marked on the graphs.

Next, we investigated the function and role of macrophages in vitro. Bone marrow–derived macrophages (BMDMs) were extracted and cultured in a hyperglycemic medium. LPS, a common agent used to induce cellular inflammatory responses, was used to activate BMDMs. GW3965 or vehicle (PBS) was added to the medium to study the role of LXR in macrophages. qPCR analysis of macrophage RNA showed that activation of LXR reduced pro‐inflammatory cytokines in macrophages, similar to in vivo experiments (Figure 4A). Immunofluorescence (IF) staining of NLRP3 showed that GW3965 inhibited NLRP3 expression in macrophages (Figure 4B,C). Western blot analysis of NLRP3, NF‐κB, and cleaved caspase‐1 (Figure 4D,E) further supported that activation of LXRs suppresses macrophage inflammatory activation.

### 3.4 LXR Agonist Attenuated Macrophage Lipid Peroxidation to Restrain Inflammation in Diabetic Liver

LXR is highly associated with lipid metabolism, and increased lipid efflux has been observed in neuroglia after being treated with an LXR agonist [12, 25]. Lipid peroxidation is well known to activate macrophage inflammation [26]. Thus, we hypothesized that the more severe inflammation in diabetic liver is due to hyperglycemia inhibiting LXR expression and exacerbating macrophage lipid peroxidation.

**Figure 5:**
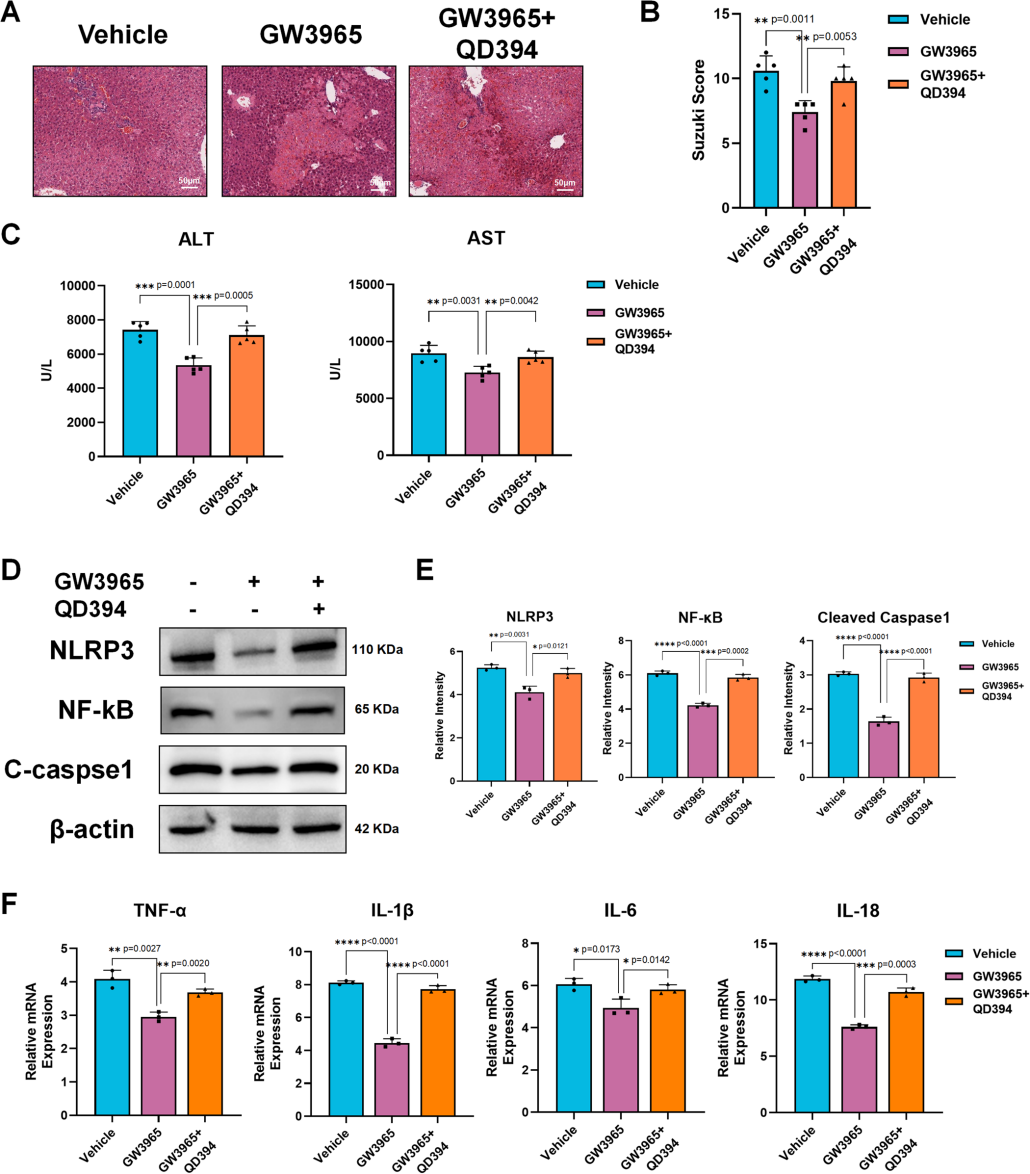
LXR agonist attenuated macrophage lipid peroxidation to restrain inflammation in diabetic liver. Diabetes C57BL/6 mice induced by STZ were pretreated with GW3965 or GW3965 + QD394 or PBS (Vehicle) before subjected to 70% liver IRI model (*n* = 5/group). (A). Representative H&E staining of liver tissue (scale bar: 50 μm) (B). Suzuki Score of different groups (*n* = 5/group). (C). ALT and AST in mice serum were tested (*n* = 5/group). (D). Western Blot detection of NLRP3, NF‐κB, cleaved caspase‐1 (C‐caspase1), and β‐actin in liver tissue (*n* = 3/group). (E). Data statistics of WB strips gray value obtained by ImageJ (*n* = 3/group). (F). Gene expression of TNF‐α, IL‐1β, IL‐6 and IL‐10 relative to GAPDH in liver tissues measured by qRT‐PCR (*n* = 3/group). *p < 0.05, **p < 0.01, ***p < 0.001, and ****p < 0.0001. *p*‐values were marked on the graphs.

SOD and MDA levels in macrophage were tested as Figure 4F showed. Higher SOD levels and lower MDA levels in macrophage treated with GW3965 suggest that the LXR agonist inhibited macrophage lipid peroxidation. Therefore, we used QD394, a lipid peroxidation activator, to conduct a rescue experiment in vivo. H&E staining and Suzuki scores showed that using QD394 prevented the protective effect of GW3965 (Figure 5A,B). The results of serum ALT and AST levels also supported this (Figure 5C). Western blot analysis also showed increased expression of NLRP3, NF‐κB, and cleaved caspase‐1 after treatment with QD394 (Figure 5D,E). The macrophage inflammatory response was also recovered, as evidenced by the expression of pro‐inflammatory cytokines (Figure 5F).

## 4 Discussion

In the present study, we examined the change of LXR expression in diabetes and liver IRI, and its role in regulating macrophage inflammation. We demonstrated that LXR expression was reduced in the hyperglycemia microenvironment and reduced further by liver IRI, which caused macrophage lipid peroxidation and inflammatory activation. The LXR agonist could rescue diabetic liver inflammation and alleviate IRI.

A series of reports have shown that diabetes exacerbates organ IRI. It has been reported that metformin can activate the mitophagy pathway and protect against hyperglycemic cerebral IRI [27]. In fact, many signaling pathways could regulate liver IRI in different ways. NK‐κB, an important inflammation driving factor in liver IRI, can be inhibited by HSP110 [28]. It is also reported regulator of stress signaling, such as FBXW5 and ESTAB regulate liver IRI [29, 30]. Diabetes also exacerbates myocardial infarction and IRI. The E3 ligase‐dead mutant MG53‐C14A may help improve cardiac IRI [31]. Recent research has reported that hyperglycemia impairs mitochondrial function and exacerbates liver IRI [32]. However, the mechanism by which hyperglycemia aggravates liver IRI remains unclear. The pathological changes in diabetes include metabolic (lipid) signaling, mitochondrial metabolism, endocrine dysregulation, and local inflammation [33]. The liver is the metabolic center of the whole body and plays an important role in glucose and lipid metabolism. Liver becomes more fragile and more sensitive to oxidative stress injury under the influence of diabetes [34, 35]. In our study, we found that LXR, which is vital for lipid transport and metabolism, is affected by hyperglycemia and liver IRI.

Macrophages are the most numerous nonparenchymal cells in the liver and play an essential role in regulating liver homeostasis [36]. Functional alter of macrophage is vital in inflammatory liver disease, including liver IRI. FSTL1 promotes liver inflammatory reprogramming in liver fibrosis [37]. Macrophages also mediate inflammation in metabolic dysfunction‐associated steatotic liver disease (MASLD) and acetaminophen‐induced liver injury [38, 39]. Impaired macrophage efferocytosis has been reported to aggravate liver IRI in aged individuals [40]. Hyperglycemia affects macrophage function. Macrophages in diabetic wounds showed dysregulation and persistent pro‐inflammatory (M1) polarization and influence wound healing [41]. Research on diabetic nephropathy has showed that macrophages are highly associated with disease progression [42]. In the context of a high‐glucose microenvironment, macrophage tends to shift towards pro‐inflammatory phenotypes, characterized by the production of pro‐inflammatory cytokines. This shift is often driven by the activation of the NLRP3 inflammasome. High glucose levels have been shown to enhance NLRP3 activation, thereby exacerbating the inflammation during the IRI process [43]. Macrophages play central roles in the progression of arteriosclerosis as well [44]. In the current study, we found that diabetes impaired macrophage lipid metabolism during liver IR and caused more severe aseptic inflammation (TNF‐α, IL‐1β, IL‐6, and IL‐18).

LXR was found to coordinate inflammation and metabolism. LXRs are known as receptors that regulate cholesterol metabolism as well as bile acid metabolism. They have also been found to have anti‐inflammatory functions. These functions make LXRs potential targets for diseases including lipid disorder, chronic inflammation, cancer, and neurodegenerative diseases [12]. In study of certain diseases, however, LXR‐α and LXR‐β perform unique functions. LXR‐α not only promotes cholesterol efflux but also activates the fatty acid synthesis pathway and causes liver lipid accumulation [45]. The function of LXR‐β in regulating cholesterol efflux is relatively mild. A study in humanized mice showed that LXR‐α, and not LXR‐β, is responsible for the induction of human UGT1A1 and bilirubin metabolism [46]. In Kupffer cells, ZEB2 functionally maintains the tissue‐specific identity of macrophages by regulating of LXR‐α [47]. LXR‐β can regulate the activation of inflammation, which plays a potential anti‐depressive role. These unique functions of LXR‐α and LXR‐β make the development of specific LXR agonist a trend for future.

LXR agonists have been reported to exhibit anti‐inflammatory effects in many diseases [48, 49]. LXR‐associated fatty acid metabolism reprogramming helps relieve inflammation in MASLD [50]. Macrophage efferocytosis is also related to LXR in apoptotic cell clearance and subsequent inflammation [51]. LXR signaling induces SMPDL3A, which can directly restrict cGAS‐STING signaling‐associated inflammation [24]. In studies of cerebral inflammation, 25‐Hydroxycholesterol was found to promote NLRP3 assembly via mitochondrial ROS and the LXR pathway [52]. The LXR agonist has been shown to promote mitochondrial ROS production and inhibit IL‐1β production, caspase‐1 cleavage, and ASC oligomerization via NLRP3 activation [53], consistent with our current research. To date, there is little evidence showing LXR inhibition direct activates NLRP3, however, LXR inhibition has been shown to influence NLRP3 by causing cholesterol metabolism disorder or mitochondrial injury and oxidative stress imbalance. Many studies also found that LXR regulates NLRP3 activation via lipid products or their receptors [54, 55]. In our research, we propose that lipid peroxidation could be the reason that macrophage NLRP3 activation, and that LXR agonists could prevent this alteration.

Therapeutical strategies targeting LXR have shown promising potential. However, some LXRs agonists have demonstrated a significant degree of promiscuity, targeting many other nuclear receptors that may significantly alter hepatic gene expression [56]. Research has shown that LXR activation exerts beneficial effects on glucose control in mouse models of type 2 diabetes. Thus, LXR may also play a role in metabolic diseases, such as MASLD. Overactivation of the LXR is the reason why abnormal upregulation of hepatic lipogenesis in MASLD [57, 58]. However, LXR agonists may also activate the fatty acid synthesis pathway, leading to liver lipid accumulation [45]. Thus, despite its potential therapeutic value in multiple diseases, the clinical application of LXR targeting still needs to overcome challenges related to safety, efficacy, and drug selectivity, especially in diabetic patients. What is promising is that the further study of the current mechanism could help relieve clinical liver IRI, as well as marginal donor liver transplantation. Liver IRI is the main reason of liver transplantation failure, especially for MASLD [59]. Our study focuses on inflammatory injury in the diabetic liver IRI process and identifies impaired LXR as a key factor in the exacerbation of liver IRI by diabetes and LXR agonist can mitigate liver injury in diabetic IRI. This mechanism may also applicable to patients with fatty liver.

## 5 Study Limitations

There are limitations in the current study. We utilized the STZ‐induced diabetes mouse model which is an acute model of diabetes and does not fully represent the long‐term hepatic effect of diabetes. The mechanism by which LXRs regulate macrophage inflammation requires further investigation and lipid peroxidation may serve as a promising therapeutic target. In addition, the prolonged course of diabetes is associated with lipid metabolism disorder which can alter LXR expression and the immune microenvironment. Change in this baseline factors may influence subsequent mechanistic studies in our current research.

## 6 Conclusion

To the best of our knowledge, this is the first study to report the critical role of LXR in regulating liver ischemia reperfusion injury. We demonstrated that reduced LXR expression in diabetic liver promotes macrophage lipid peroxidation and hence exacerbates inflammation which plays vital role in liver IRI. Activation of LXR could help protect liver IRI in diabetic liver. Our results contribute to understanding the function of macrophages in liver IRI and provide a new target, LXR, for prevention and treatment of liver IRI in patients with diabetes. Research on LXR intervention drugs may be a promising direction in the future.

## Author Contributions

**Chuanwei Jiang:** validation, investigation, methodology, supervision, writing – review and editing, data curation. **Wenzhou Ding:** writing – review and editing, methodology, validation, supervision, formal analysis, visualization. **Yuanchang Hu:** methodology, validation, formal analysis, visualization, writing – review and editing. **Chao Yang:** conceptualization, methodology, writing – original draft, writing – review and editing, investigation, supervision, project administration, data curation.

## Ethics Statement

The study protocol was approved by the Institutional Review Board of the First Affiliated Hospital with Nanjing Medical University. All animal procedures met the relevant legal and ethical requirements according to the protocol (2024‐SRFA‐649) endorsed by the Institutional Animal Care and Use Committee of Nanjing Medical University in accordance with ARRIVE guidelines.

## Conflicts of Interest

The authors declare no conflicts of interest.

## Data Availability

The data that support the findings of this study are available from the corresponding author upon reasonable request. All data generated or analyzed during this study are included in this published article.
